# Effects of Mulching Tolerant Plant Straw on Soil Surface on Growth and Cadmium Accumulation of *Galinsoga parviflora*

**DOI:** 10.1371/journal.pone.0114957

**Published:** 2014-12-09

**Authors:** Lijin Lin, Ming’an Liao, Yajun Ren, Li Luo, Xiao Zhang, Daiyu Yang, Jing He

**Affiliations:** 1 College of Horticulture, Sichuan Agricultural University, Ya’an, Sichuan 625014, China; 2 Ya’an Soil and Water Conservation Monitoring Substation, Ya’an, Sichuan 625000, China; INRA, France

## Abstract

Pot and field experiments were conducted to study the effects of mulching with straw of cadmium (Cd) tolerant plants (*Ranunculus sieboldii*, *Mazus japonicus*, *Clinopodium confine* and *Plantago asiatica*) on growth and Cd accumulation of *Galinsoga parviflora* in Cd-contaminated soil. In the pot experiment, mulching with *M. japonicus* straw increased the root biomass, stem biomass, leaf biomass, shoot biomass, plant height and activities of antioxidant enzymes (superoxide dismutase, peroxidase and catalase) of *G. parviflora* compared with the control, whereas mulching with straws of *R. sieboldii*, *C. confine* and *P. asiatica* decreased these parameters. Straws of the four Cd-tolerant plants increased the Cd content in roots of *G. parviflora* compared with the control. However, only straws of *M. japonicus* and *P. asiatica* increased the Cd content in shoots of *G. parviflora*, reduced the soil pH, and increased the soil exchangeable Cd concentration. Straw of *M. japonicus* increased the amount of Cd extraction in stems, leaves and shoots of *G. parviflora* by 21.11%, 29.43% and 24.22%, respectively, compared with the control, whereas straws of the other three Cd-tolerant plants decreased these parameters. In the field experiment, the *M. japonicus* straw also increased shoot biomass, Cd content in shoots, and amount of Cd extraction in shoots of *G. parviflora* compared with the control. Therefore, straw of *M. japonicus* can be used to improve the Cd extraction ability of *G. parviflora* from Cd-contaminated soil.

## Introduction

As anthropogenic impacts on the environment continue to intensify, increasing soil contamination with heavy metals has become a major factor harming human health [1]. In China, the area of cadmium (Cd) contaminated soil has increased to 200,000 km^2^, which is one-sixth of the arable land area [2]. Therefore, the heavy-metal-contaminated soils require immediate treatment, especially for Cd. A variety of methods are suitable for remediation of heavy-metal-contaminated soil, of which phytoremediation is a commonly used method [3]. Phytoremediation maintains the soil structure and microbial community, while the roots of hyperaccumulator plants directly absorb heavy metals from the soil and transfer them to the aboveground shoots to achieve remediation of contaminated soil [4]. However, the main hyperaccumulator materials used for phytoremediation are slow-growing and produce low biomass, thus large-scale application is difficult [5]–[6]. Therefore, improvement in the remediation ability of hyperaccumulator is of practical importance.

Methods that can improve the remediation ability of hyperaccumulators have been screened, such as application of surfactant chelator to the soil [7], intercropping [8]–[9], and spray application of a plant growth regulator [10]–[11]. While these measures have been beneficial to a certain degree, their application has not been widely implemented. As diverse agricultural production practices are used in different areas, screening of more natural methods to improve remediation ability is necessary. Straw mulching is a technique that is commonly used in agricultural production. The main purpose of the straw mulch is the conversion of plant straw into organic matter and other nutrients, and to fertilize the soil and improve soil texture [12]–[13]. The straw can improve the physical and chemical properties of soil, increase soil organic matter content, and therefore promote crop growth, increase yield, and improve crop quality [14]. These effects are related to the nutrients and allelochemicals released from the straw. Release of allelochemicals is a form of allelopathy, which can affect soil nutrient availability, soil enzyme activity, microbial population structure, and plant growth [15]. If plant straw was applied to heavy metal-contaminated soil, allelochemicals may regulate hyperaccumulator growth and heavy metal accumulation. As Cd-tolerant plants, *Ranunculus sieboldii*, *Clinopodium confine*, *Mazus japonicus* and *Plantago asiatica* show high tolerance to Cd stress, and Cd contents in the shoots of these species are much lower than those of a hyperaccumulator or accumulator [16]. In this study, we grew seedlings of the Cd hyperaccumulator *Galinsoga parviflora* [16] in Cd-contaminated soil. Shoots of the above-mentioned Cd-tolerant species were applied as mulches on the soil surface to screen their efficiency at promoting the growth and Cd accumulation of *G. parviflora*, and to provide materials for improvement in the phytoremediation ability of *G. parviflora*.

## Materials and Methods

### Ethics statement

The field study was conducted in an area where *Ranunculus sieboldii*, *Clinopodium confine*, *Mazus japonicus*, *Plantago asiatica* and *Galinsoga parviflora* were the main weeds. This site was on the Ya'an campus of Sichuan Agricultural University Farm (29°59′N, 102°59′E), Ya’an City, Sichuan Province, China. The experimental field belongs to Sichuan Agricultural University, which is authorized by the government of Ya'an City. The field is not privately owned or protected. No specific permits were required for the described field studies. During the experiment, no other specific permissions were required because we conducted normal agricultural activities and no endangered or protected species were involved.

### Plant and soil materials

In August 2013, shoots of *Ranunculus sieboldii*, *Clinopodium confine*, *Mazus japonicus* and *Plantago asiatica* were collected from the Ya’an campus farm of the Sichuan Agricultural University (29° 59′ N, 102° 59′ E), China, at sites where the soil was not contaminated by heavy metals. The shoots were dried at 80°C to constant weight, then finely ground and sieved through a 5-mm-mesh nylon sieve. *Galinsoga parviflora* seedlings with two pairs of euphyllas were collected from the Ya’an campus farm at a site not contaminated by heavy metals in September 2013.

Inceptisol soil samples (purple soil in the Genetic Soil Classification of China) were collected from the Ya’an campus farm in August 2013. The basic chemical properties of the soil were pH 7.02, organic matter 41.38 g kg^−1^, total nitrogen (N) 3.05 g kg^−1^, total phosphorus (P) 0.31 g kg^−1^, total potassium (K) 15.22 g kg^−1^, alkali soluble N 165.30 mg kg^−1^, available P 5.87 mg kg^−1^, and available K 187.03 mg kg^−1^. The total Cd content was 0.101 mg kg^−1^ and the available Cd content was 0.021 mg kg^−1^. The basic soil properties and heavy metal concentrations were determined according to Bao (2000) [17].

### Pot experiment

The experiment was conducted at the Ya’an campus farm in August–October 2013. The soil samples were air-dried and passed through a 5-mm sieve in August 2013, and then 4.0 kg of the air-dried soil was weighed into each polyethylene pot (15 cm high, 18 cm diameter). Cadmium was added to the soil samples as CdCl_2_·2.5 H_2_O at 10 mg kg^−1^ [18]. The pots were soaked in the Cd solution for 4 weeks, and then the soil in each pot was mixed thoroughly. Five uniform seedlings of *G. parviflora* were transplanted into each pot, and 6 g shoots [19] of four Cd-tolerant species were applied as mulches on the soil surface in each pot (equivalent to 225 g m^−2^). Five treatments were applied: not mulched with straw (control), mulched with *R. sieboldii* straw, mulched with *C. confine* straw, mulched with *M. japonicus* straw, and mulched with *P. asiatica* straw. Each treatment was repeated three times with a completely randomized design with 10-cm spacing between pots. The soil moisture was maintained at 80% of field capacity from when the *G. parviflora* plants were transplanted until the plants were harvested.

At maturity (after 50 d), the height of the *G. parviflora* plants was measured, and the uppermost young leaves of 2 cm in length were collected to determine the activities of the antioxidant enzymes superoxide dismutase (SOD), peroxidase (POD), and catalase (CAT) [20]. Then, the entire plants were harvested. The roots, stems, and leaves were washed with tap water, and the roots were immersed in 10 mM L^−1^ HCl for 10 min to remove Cd adhering to the root surface. The roots, stems, and leaves were further washed with deionized water and dried at 80°C to constant weight for dry weight and Cd content determination. The dried plant samples were finely ground and sieved through a 0.149-mm-mesh nylon sieve for chemical analysis. Samples (0.5 g) were digested in HNO_3_/HClO_4_ (4∶1, v/v), and then the volume was diluted to 50 ml with deionized water. The Cd concentrations in roots, stems, and leaves were determined using an iCAP 6300 ICP spectrometer (Thermo Scientific, Waltham, MA, USA) [17]. The pot soils were dried naturally and ground into powder (granule diameter <1 mm) to determine the pH and exchangeable Cd concentration. Soil pH was measured in a 1∶2.5 (w/v) suspension of soil and deionized water, and the exchangeable Cd in the soil was extracted with 0.005 mol L^−1^ DTPA-TEA and analyzed with an iCAP 6300 ICP spectrometer [17].

### Field experiment

The field experiment was conducted at the Ya’an campus farm in February–March 2014. Inceptisol soil samples were collected from the Cd-contaminated area of the farm. The basic chemical properties of the soil were as follows: pH 6.96, organic matter 34.33 g kg^−1^, total N 1.14 g kg^−1^, total P 0.66 g kg^−1^, total K 21.54 g kg^−1^, alkali solution N 78.55 mg kg^−1^, available P 33.67 mg kg^−1^, available K 111.07 mg kg^−1^, and total Cd 1.80 mg kg^−1^. The basic soil properties and heavy metal concentrations were determined according to Bao (2000) [17]. Each plot area was 1.0 m^2^ (1.0 m×1.0 m). Seedlings of *G. parviflora* were planted directly in the soil at a density of 100 plants m^−2^ (in a 10 cm×10 cm grid) in February 2014. Shoots of the four Cd-tolerant species were applied as mulches (density 225 g m^−2^) on the soil surface in each plot. The five treatments in the experiment were identical to those of the pot experiment. Each treatment was repeated three times (three plots). The cultivation and management of *G. parviflora* seedlings were as previously described for the pot experiment. At maturity (after 50 d), the *G. parviflora* shoots were harvested to determine shoot biomass and Cd content as described for the pot experiment.

### Statistical analyses

Statistical analyses were performed using SPSS 13.0 statistical software (IBM, Chicago, IL, USA). Data were analyzed by one-way ANOVA with least significant difference (LSD) at the 5% confidence level.

The following ratios were calculated: root/shoot ratio = root biomass/shoot biomass [21]; shoot bioconcentration factor (BCF) = Cd content in shoot/Cd concentration in soil [22]; translocation factor (TF) = Cd content in shoot/Cd content in root [23]; translocation accumulation factor (TAF) = Cd content in shoot×shoot biomass)/Cd content in root×root biomass [24].

## Results

### Biomass of *G. parviflora*

In the pot experiment, the *M. japonicus* straw mulch significantly (*P*<0.05) increased the root, stem, leaf, and shoot biomasses of *G. parviflora* by 18.75%, 9.35%, 33.33%, and 16.11%, respectively, compared with those of the control, whereas mulching with *R. sieboldii*, *C. confine*, and *P. asiatica* straws decreased these biomasses compared with the control (Table 1). The treatment effects on root biomass of *G. parviflora* were ranked as *M. japonicus* straw > control >*P. asiatica* straw > *C. confine* straw > *R. sieboldii* straw, and on stem, leaf and shoot biomass as *M. japonicus* straw > control > *C. confine* straw >*P. asiatica* straw > *R. sieboldii* straw. Mulching with straws of the four Cd-tolerant species increased the root/shoot ratio of *G. parviflora* compared with the control, for which the treatment effects were ranked as *P. asiatica* straw > *R. sieboldii* straw > *C. confine* straw > *M. japonicus* straw > control (Table 1), which indicated that straws of the four Cd-tolerant species promoted *G. parviflora* root growth.

**Table 1:** Biomass of *G. parviflora* in the pot experiment.

Cadmium-tolerant species	Roots (g plant^−1^)	Stems (g plant^−1^)	Leaves (g plant^−1^)	Shoots (g plant^−1^)	Root/shoot ratio
Control	0.48±0.05b	1.07±0.05b	0.42±0.02b	1.49±0.07b	0.32
*R. sieboldii*	0.40±0.03d	0.70±0.01c	0.27±0.04d	0.97±0.05d	0.41
*C. confine*	0.42±0.02cd	0.72±0.01c	0.35±0.03c	1.07±0.04c	0.39
*M. japonicus*	0.57±0.03a	1.17±0.03a	0.56±0.04a	1.73±0.07a	0.33
*P. asiatica*	0.46±0.01bc	0.71±0.03c	0.32±0.01c	1.03±0.04cd	0.45

Plants were cultured in soil containing 10 mg added Cd (kg soil)^−1^ for 50 d. Values are means (±SE) of three replicate pots. Different lowercase letters within a column indicate significant differences based on one-way analysis of variance in SPSS 13.0 followed by the least significant difference test (*P*<0.05). The root/shoot ratio = root biomass/shoot biomass.

### Plant height of *G. parviflora*

**Figure 1:**
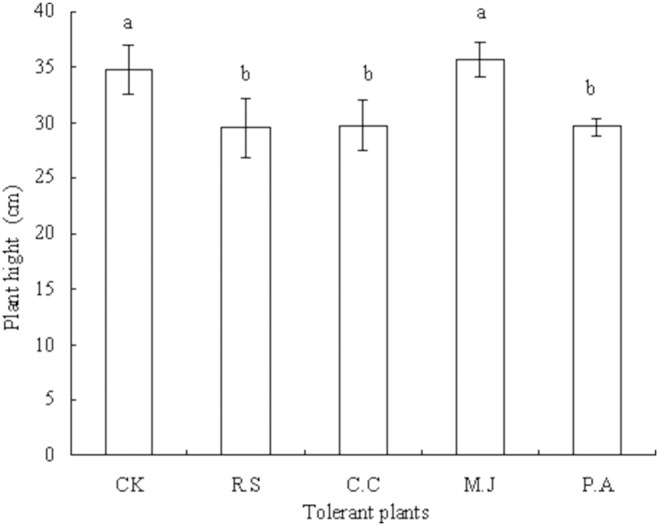
Height of *G. parviflora* plants in the pot experiment. Plants were cultured in soil containing 10 mg added Cd (kg soil)^−1^ for 50 d. Values are means of three replicate pots. Different lowercase letters indicate significant differences based on one-way analysis of variance in SPSS 13.0 followed by the least significant difference test (*P*<0.05). CK = control, R.S = *Ranunculus sieboldii*, C.C = *Clinopodium confine*, M.J = *Mazus japonicus*, P.A = *Plantago asiatica*.

As for *G. parviflora* biomass, in the pot experiment *M. japonica* straw increased the plant height of *G. parviflora* by 2.59% (*P*>0.05), whereas *R. sieboldii*, *C. confine*, and *P. asiatica* straws decreased the plant height by 15.23% (*P*<0.05), 14.66% (*P*<0.05), and 14.94% (*P*<0.05), respectively, compared with the control (Fig. 1).

### Antioxidant enzyme activity of *G. parviflora*

*Mazus japonicus* straw enhanced the activities of SOD, POD and CAT of *G. parviflora* in the pot experiment by 8.31% (*P*<0.05), 9.42% (*P*<0.05), and 6.68% (*P*<0.05), respectively, compared with the control, whereas *R. sieboldii*, *C. confine*, and *P. asiatica* straws decreased activities of these enzymes (Table 2). The treatment effects on SOD, POD and CAT activities of *G. parviflora* were ranked as *M. japonicus* straw > control > *C. confine* straw >*P. asiatica* straw > *R. sieboldii* straw, which was consistent with the effects on *G. parviflora* biomass.

**Table 2:** Antioxidant enzyme activities of *G. parviflora* in the pot experiment.

Cadmium-tolerant species	SOD activity	POD activity	CAT activity
	(U·g^−1^ FW·h^−1^)	(µg·g^−1^ FW·min^−1^)	(U·g^−1^ FW·min^−1^)
Control	337.21±11.53b	279.80±5.20b	51.63±0.49b
*R. sieboldii*	279.78±3.16d	204.72±8.72d	45.12±0.61d
*C. confine*	312.46±11.29c	256.05±7.95c	50.41±1.22bc
*M. japonicus*	365.22±8.40a	306.15±9.85a	55.08±1.39a
*P. asiatica*	292.65±9.32d	251.86±6.86c	48.77±1.73c

Superoxide dismutase (SOD), peroxidase (POD), and catalase (CAT) activities were measured in plants cultured for 50 d in soil containing 10 mg added Cd (kg soil)^−1^. Values are means (±SE) of three replicate pots. Different lowercase letters within a column indicate significant differences based on one-way analysis of variance in SPSS 13.0 followed by the least significant difference test (*P*<0.05).

### Cadmium content in *G. parviflora*

Mulching with straws of the four Cd-tolerant species changed the Cd distribution in organs of *G. parviflora* in the pot experiment. All of the straws increased the Cd content in roots of *G. parviflora* in the rank order *P. asiatica* straw > *M. japonicus* straw > *R. sieboldii* straw > *C. confine* straw > control (Table 3). Only the straws of *M. japonicus* and *P. asiatica* increased the Cd content in stems of *G. parviflora*, which increased by 10.75% (*P*<0.05) and 4.64% (*P*>0.05), respectively, compared with that of the control, whereas the straws of *R. sieboldii* and *C. confine* decreased the stem Cd content. The treatments affected Cd content in leaves of *G. parviflora* in the rank order *P. asiatica* straw > *R. sieboldii* straw > *C. confine* straw > *M. japonicus* straw > control. The treatments had a similar effect on Cd content in shoots of *G. parviflora* to that on stem Cd content. The straws of *M. japonicus* and *P. asiatica* increased shoot Cd content by 8.34% (*P*<0.05) and 13.67% (*P*<0.05), respectively, compared with the control. The shoot BCF of *G. parviflora* was enhanced by the straws of *M. japonicus* and *P. asiatica*, but was decreased by the straws of *R. sieboldii* and *C. confine*, compared with the control (Table 3). The straw of *M. japonica* enhanced the TF of *G. parviflora*, whereas the other straw types decreased the TF, compared with that of the control.

**Table 3:** Cadmium content in *G. parviflora* in the pot experiment.

Cadmium-tolerant species	Roots (mg kg^−1^)	Stems (mg kg^−1^)	Leaves(mg kg^−1^)	Shoots (mg kg^−1^)	ShootBCF	TF
Control	20.10±1.07b	21.12±1.07b	32.12±1.06b	24.22±1.10b	2.42	1.20
*R. sieboldii*	21.21±1.56b	18.53±0.99c	38.18±2.42a	24.00±2.02b	2.40	1.13
*C. confine*	20.77±1.12b	18.15±1.15c	32.38±2.16b	22.80±0.21b	2.28	1.10
*M. japonicus*	21.65±0.95b	23.39±1.65a	31.18±1.36b	26.24±0.62a	2.62	1.21
*P. asiatica*	24.48±1.68a	22.10±1.26ab	39.57±1.07a	27.53±0.89a	2.75	1.12

Plants were cultured in soil containing 10 mg added Cd (kg soil)^−1^ for 50 d. Values are means (±SE) of three replicate pots. Different lowercase letters within a column indicate significant differences based on one-way analysis of variance in SPSS 13.0 followed by the least significant difference test (*P*<0.05). The translocation factor (TF) is defined as Cd content in shoot/Cd content in root and the shoot bioconcentration factor (BCF) is defined as Cd content in shoot/Cd concentration in soil.

### Cadmium extraction by *G. parviflora*

In the pot experiment, the straws of *M. japonicus* and *P. asiatica* increased the amount of Cd extraction in roots of *G. parviflora* by 27.88% (*P*<0.05) and 16.68% (*P*<0.05), respectively, compared with that of the control, whereas the *R. sieboldii* and *C. confine* straws decreased root Cd extraction (Table 4). Only *M. japonicus* straw increased the amount of Cd extraction in stems, leaves, and shoots of *G. parviflora* by 21.11% (*P*<0.05), 29.43% (*P*<0.05), and 24.22% (*P*<0.05), respectively, compared with the control, whereas the other three straw types decreased Cd extraction in these organs (Table 4). The treatment effects on the TAF of *G. parviflora* were ranked as control > *M. japonicus* straw > *C. confine* straw > *R. sieboldii* straw >*P. asiatica* straw.

**Table 4:** Cadmium extraction by *G. parviflora* in the pot experiment.

Cadmium-tolerant species	Roots (µg plant^−1^)	Stems (µg plant^−1^)	Leaves (µg plant^−1^)	Shoots (µg plant^−1^)	TAF
Control	9.65±1.53c	22.60±2.11b	13.49±1.24b	36.09±3.35b	3.74
*R. sieboldii*	8.48±1.30d	12.97±0.56d	10.31±2.22d	23.28±2.78d	2.75
*C. confine*	8.72±0.01cd	13.07±0.99d	11.33±1.58cd	24.40±2.57d	2.80
*M. japonicus*	12.34±0.15a	27.37±2.71a	17.46±0.47a	44.83±3.18a	3.63
*P. asiatica*	11.26±0.51b	15.69±1.52c	12.66±0.22bc	28.35±1.74c	2.52

Plants were cultured in soil containing 40 mg added Cd (kg soil)^−1^ for 50 d. Values are means (±SE) of three replicate pots. Different lowercase letters within a column indicate significant differences based on one-way analysis of variance in SPSS 13.0 followed by the least significant difference test (*P*<0.05). Translocation accumulation factor (TAF) = (Cd content in shoot×shoot biomass)/Cd content in root×root biomass.

### Soil pH and soil exchangeable Cd

**Figure 2:**
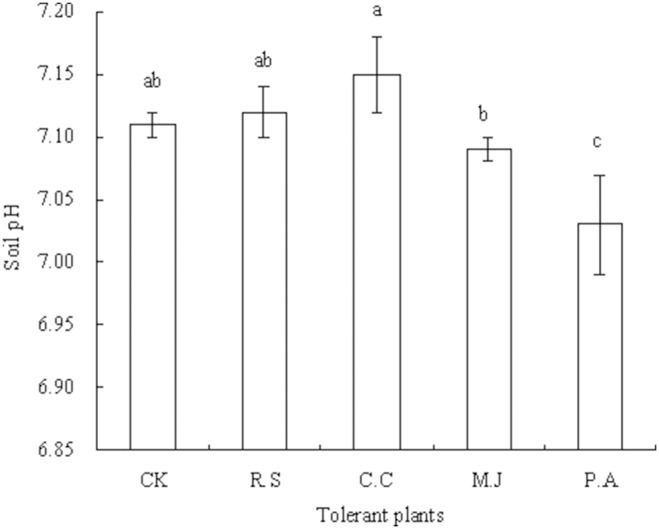
Soil pH in the pot experiment. Plants were cultured in soil containing 10 mg added Cd (kg soil)^−1^ for 50 d. Values are means of three replicate pots. Different lowercase letters indicate significant differences based on one-way analysis of variance in SPSS 13.0 followed by the least significant difference test (*P*<0.05). CK = control, R.S = *Ranunculus sieboldii*, C.C = *Clinopodium confine*, M.J = *Mazus japonicus*, P.A = *Plantago asiatica*.

**Figure 3:**
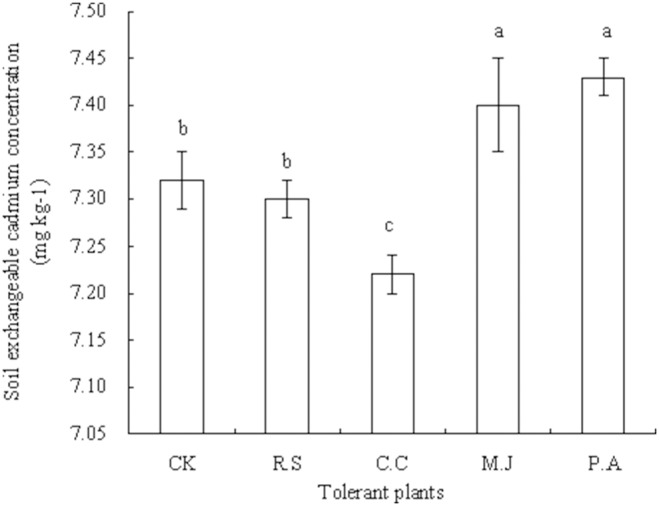
Soil exchangeable cadmium in the pot experiment. Plants were cultured in soil containing 10 mg added Cd (kg soil)^−1^ for 50 d. Values are means of three replicate pots. Different lowercase letters indicate significant differences based on one-way analysis of variance in SPSS 13.0 followed by the least significant difference test (*P*<0.05). CK = control, R.S = *Ranunculus sieboldii*, C.C = *Clinopodium confine*, M.J = *Mazus japonicus*, P.A = *Plantago asiatica*.

The straws of *R. sieboldii* and *C. confine* increased the soil pH (*P*>0.05) compared with that of the control, whereas straws of *M. japonicus* (*P*>0.05) and *P. asiatica* (*P*<0.05) decreased soil pH in the pot experiment (Fig. 2). The treatment effects on soil pH were ranked as *C. confine* straw > *R. sieboldii* straw > control > *M. japonicus* straw >*P. asiatica* straw. The effects on soil exchangeable Cd concentration were ranked as *P. asiatica* straw > *M. japonicus* straw > control > *R. sieboldii* straw > *C. confine* straw in the pot experiment (Fig. 3). The straws of *R. sieboldii* and *C. confine* decreased the soil exchangeable Cd concentration by 0.27% (*P*>0.05) and 1.37% (*P*<0.05), respectively, whereas the straws of *M. japonicus* and *P. asiatica* increased soil exchangeable Cd concentration by 1.09% (*P*<0.05) and 1.50% (*P*<0.05), respectively, compared with that of the control.

### Field experiment

In the field experiment, *M. japonicus* straw increased the shoot biomass of *G. parviflora* compared with that of the control (*P*<0.05), whereas the other three treatments decreased the shoot biomass (Table 5), which was consistent with the results of the pot experiment. Mulching with the straws of *M. japonicus* and *P. asiatica* increased, whereas the straws of *R. sieboldii* and *C. confine* decreased the Cd content in shoots of *G. parviflora* compared with that of the control. Compared with the control, the Cd extraction amount in shoots of *G. parviflora* was increased by *M. japonicus* straw by 6.41% (*P*<0.05), but was decreased by the other three treatments (Table 5).

**Table 5:** Biomass and cadmium accumulation in the field experiment.

Cadmium-tolerant species	Shoot biomass (g m^−2^)	Cd content in shoots (mg kg^−1^)	Cd extraction by shoots (mg m^−2^)
Control	478.14±10.49b	3.27±0.07a	1.56±0.06b
*R. sieboldii*	447.68±12.90d	3.15±0.11b	1.41±0.01c
*C. confine*	467.59±10.72bc	3.08±0.10b	1.44±0.08c
*M. japonicus*	497.24±9.45a	3.33±0.08a	1.66±0.01a
*P. asiatica*	459.68±10.35cd	3.35±0.04a	1.54±0.01b

Plants were cultured in soil containing 1.8 mg added Cd (kg soil)^ −1^ for 60 d in a field experiment. Values are means (±SE) of three replicate plots. Different lowercase letters within a column indicate significant differences based on one-way analysis of variance in SPSS 13.0 followed by the least significant difference test (*P*<0.05).

## Discussion

Plant straw contains many nutrients and allelochemicals, which are released during the process of straw decay and decomposition and can be absorbed by living plants [25]. However, when plant straw decays and decomposes, organic acids are produced and can inhibit the growth of living plant roots to some extent, eventually leading to inhibition of plant shoot growth [26]. In the present pot experiment, mulching with *M. japonicus* straw increased the root, stem, leaf, and shoot biomasses of *G. parviflora* compared with those of the control, whereas the straws of *R. sieboldii*, *C. confine*, and *P. asiatica* decreased these biomasses. An identical effect on shoot biomass of *G. parviflora* was observed in the field experiment. There might be two reasons for this phenomenon. First, the promotive effect of nutrients from *M. japonica* straw on growth of *G. parviflora* exceeded the inhibitory effect of organic acids from *M. japonica* straw, whereas the promotive effect of nutrients from *R. sieboldii*, *C. confine*, and *P. asiatica* straws on growth of *G. parviflora* was weaker than the inhibitory effect of organic acids from these straws. Second, allelochemicals from *M. japonica* straw might promote growth of *G. parviflora*, whereas allelochemicals from *R. sieboldii*, *C. confine*, and *P. asiatica* straws might inhibit growth of *G. parviflora*. Ultimately, in the pot experiment, *M. japonicus* straw enhanced, whereas *R. sieboldii*, *C. confine*, and *P. asiatica* straws decreased the plant height of *G. parviflora* compared with that of the control.

Under stress conditions, plants produce O^−^_2_· in their organs, leading to cellular peroxide poisoning and damage, which inhibits plant growth and reduces plant biomass [27]. The antioxidant enzyme system (SOD, POD, and CAT) plays an important role in the protection of cells and organs from stress-related damage [28]. In the pot experiment, the straw of *M. japonicus* enhanced SOD, POD, and CAT activities of *G. parviflora* compared with that of the control, whereas the straws of *R. sieboldii*, *C. confine*, and *P. asiatica* reduced activities of these enzymes. This finding might be related to allelochemicals from *M. japonica* straw, which could enhance the tolerance of *G. parviflora* to Cd stress, whereas allelochemicals from *R. sieboldii*, *C. confine*, and *P. asiatica* straws might reduce the Cd stress tolerance of *G. parviflora*.

Under heavy metal contamination of the soil, studies on the effects of heavy metal absorption by plants have focused mainly on the rhizosphere environment [29]–[30]. Organic acids and other organic matter such as humus derived from the straw through decay and decomposition can change the rhizosphere pH, redox potential, and nutrient availability, and thereby affect the bioavailability of heavy metals in the rhizosphere soil [31]–[32]. In the current pot experiment, the soil pH was increased by the straws of *R. sieboldii* and *C. confine*, but decreased by the straws of *M. japonicus* and *P. asiatica* compared with that of the control. It may be that allelochemicals from *R. sieboldii* and *C. confine* straws promote the secretion of organic acids from the roots of *G. parviflora* during plant growth. In addition, in the pot experiment, the soil exchangeable Cd concentration was decreased by the straws of *R. sieboldii* and *C. confine*, and increased by the straws of *M. japonicus* and *P. asiatica*.

The straws of *R. sieboldii*, *C. confine*, *M. japonicus*, and *P. asiatica* increased the Cd content in roots of *G. parviflora* compared with that of the control in the pot experiment, but only the straws of *M. japonicus* and *P. asiatica* increased the Cd content in shoots of *G. parviflora* in both the pot and field experiments. This result indicates that the organic acids and other organic matter derived from the straw by decay and decomposition can lead to increased Cd content in roots of *G. parviflora*, and only allelochemicals from *M. japonica* straw may increase the Cd content in shoots of *G. parviflora*. Thus, allelochemicals from different plant species may play different roles in Cd absorption and translocation by *G. parviflora*. The straws of *M. japonicus* and *P. asiatica* increased, whereas *R. sieboldii* and *C. confine* straws decreased, the amount of Cd extraction in roots of *G. parviflora* in the pot experiment compared with the control. Only *M. japonicus* straw increased the amount of Cd extraction in stems, leaves and shoots of *G. parviflora* compared with those of the control, whereas the other three straw types decreased Cd extraction in these organs, in both the pot and field experiments. Thus, the present results showed that the straw of *M. japonicus* can improve the phytoremediation ability of *G. parviflora* and can be used to improve the Cd extraction ability of *G. parviflora* from Cd-contaminated soil.
